# Stage-Specific Lipidomes of *Gastrodia elata* Extracellular Vesicles Modulate Fungal Symbiosis

**DOI:** 10.3390/ijms26178611

**Published:** 2025-09-04

**Authors:** Siyu Hao, Zhongyi Hua, Yuan Yuan

**Affiliations:** 1 State Key Laboratory for Quality Ensurance and Sustainable Use of Dao-di Herbs, National Resource Center for Chinese Materia Medica, China Academy of Chinese Medical Sciences, Beijing 100700, China; 18875060516@163.com; 2 Experimental Research Center, China Academy of Chinese Medical Sciences, Beijing 100700, China; 3 School of Pharmacy, Jiangsu University, Zhenjiang 212013, China

**Keywords:** extracellular vesicles, *Gastrodia elata*, lipidomics, tuber development

## Abstract

The mycoheterotrophic orchid *Gastrodia elata* relies entirely on symbiosis with *Armillaria* for nutrient acquisition during tuber development. The signaling mechanisms underlying this interaction have long been a research focus, and several pathways, such as phytohormone-mediated signaling, have been reported. However, the role of plant-derived extracellular vesicles (PDEVs) in *G. elata*–*Armillaria* communication remains unexplored. In this study, we conducted a comprehensive lipidomic analysis of *G. elata*-derived extracellular vesicles (GDEVs) isolated from juvenile, immature (active symbiosis), and mature tubers. By employing high-resolution mass spectrometry and advanced statistical methods, we established a detailed EV lipidome profile for *G. elata*, identifying 996 lipid species spanning eight major classes. Distinct lipidomic remodeling was observed throughout tuber maturation. Notably, as the immature stage corresponds to the period of peak symbiotic activity, targeted lipidome comparisons enabled the identification of core lipid markers, particularly Glc-sitosterols and the polyketide 7,8-dehydroastaxanthin, which are highly enriched during active symbiosis and potentially associated with inter-kingdom communication. These findings suggest that developmentally regulated lipid transport via EVs plays a critical role in mediating *G. elata*–*Armillaria* interaction. Our work not only illuminates the contribution of vesicle lipids to plant–fungal interaction but also provides a methodological foundation for investigating EV-mediated signaling in non-model plant–microbe systems.

## 1. Introduction

*Gastrodia elata* is a mycoheterotrophic orchid that relies entirely on fungal symbionts for its growth and development throughout its life cycle [1]. While *G. elata* seed germination requires associations with *Mycena*, *Armillaria* is the only nutrient source during the *G. elata* vegetative growth stage [2]. This obligatory interaction renders *G. elata*–*Armillaria* symbiosis a compelling model system to study molecular adaptations in mycoheterotrophic plants. Current research has primarily focused on nutrient transfer, immune modulation, and signal regulation, with particular attention paid to phytohormone-mediated communication. For instance, strigolactones, a class of plant hormones, are known to facilitate the establishment of symbiotic interactions by acting as signaling molecules to attract *Armillaria* hyphae [3]. Despite these advances, the precise mechanisms underlying symbiotic signaling, especially extracellular vesicle (EV)-mediated communication, remain poorly understood.

Plant-derived extracellular vesicles (PDEVs), as phospholipid bilayer nanoparticles (50–500 nm) [4], have emerged as crucial mediators in both intracellular communication and interspecies interactions [5]. Current research reveals their multifunctional roles extending across plant development [6], stress responses [7], and plant–microbe interactions [8,9,10]. In *Arabidopsis*, PDEVs transport small RNAs between root cells to coordinate developmental responses [6]. Concurrently, these vesicles contribute to abiotic stress adaptation through regulated molecular delivery, exemplified by tomato PDEVs that modulate miR-9476 levels to maintain cellular zinc homeostasis under salt stress [7]. Beyond their fundamental roles in plant physiology, PDEVs have emerged as fascinating players in cross-kingdom communication [8]. In arbuscular mycorrhizal (AM) symbiosis, PDEVs are enriched at the peri-arbuscular interface, suggesting their involvement in bidirectional signaling between the host and fungus [9,10]. Conversely, in pathogenic interactions, PDEVs exhibit antifungal properties through mechanisms such as interfering with fungal development. For example, *Arabidopsis* PDEVs significantly impair germ tube elongation and hyphal morphology during fungal invasion [11]. However, while the role of EVs has been documented in pathogenic and mutualistic interactions, their involvement in *G. elata*–*Armillaria* symbiosis remains unexplored.

The functional diversity of PDEVs stems from their molecular cargo, including proteins, RNAs, and lipids [12]. PDEVs carry functionally diverse proteins that mediate critical biological processes, such as annexins for membrane repair [6], 14-3-3 proteins for signal transduction [13], and pathogenesis-related proteins for plant defense [14]. Similarly, RNA cargo, such as tomato PDEVs delivering miR482b to silence *Botrytis cinerea* virulence genes [15], demonstrates the active role of PDEVs in inter-organismal communication. Lipids constitute the structurally and functionally most diverse class of biomolecules in PDEVs, accounting for over 30% of its mass [14]. These compounds play crucial roles in membrane dynamics and signaling processes. In plant–microbe interactions, specific lipids such as sphingolipids have been implicated in fungal recognition and immune evasion [16]. For instance, lipochitooligosaccharides (LCOs) secreted by AM fungi are critical for symbiotic signaling and are structurally related to certain lipid classes [17]. Given the importance of lipids in symbiotic crosstalk, a comprehensive lipidomic analysis of *G. elata*-derived EVs (GDEVs) could reveal novel lipid mediators essential for maintaining *G. elata*–*Armillaria* symbiosis.

To address this knowledge gap, this study conducted a comprehensive lipidomic analysis of GDEVs at three developmental stages, juvenile, immature, and mature, with particular emphasis on the immature stage, owing to its high symbiotic activity. GDEVs were isolated via ultracentrifugation and characterized using Q-TOF MS-based lipidomics to profile their lipid composition across stages. Comparative analyses were performed to identify core lipid markers potentially involved in symbiotic processes. This study aimed to determine whether the lipid class distribution in EVs of *G. elata*, a fully mycoheterotrophic plant, differs from that in photosynthetic species, and to evaluate the potential lipids associated with EV-mediated symbiotic interactions. We hypothesized that the lipid composition of GDEVs varies significantly across developmental stages and differs from that of photosynthetic species, with specific lipid classes playing key roles in symbiotic interactions. This work provides a lipid atlas of EVs in *G. elata* and establishes a foundational framework for research on EV-mediated communication in non-model symbiotic systems.

## 2. Results

### 2.1. Isolation and Morphological Characterization of Extracellular Vesicles from Different Developmental Stages of G. elata

**Figure 1:**
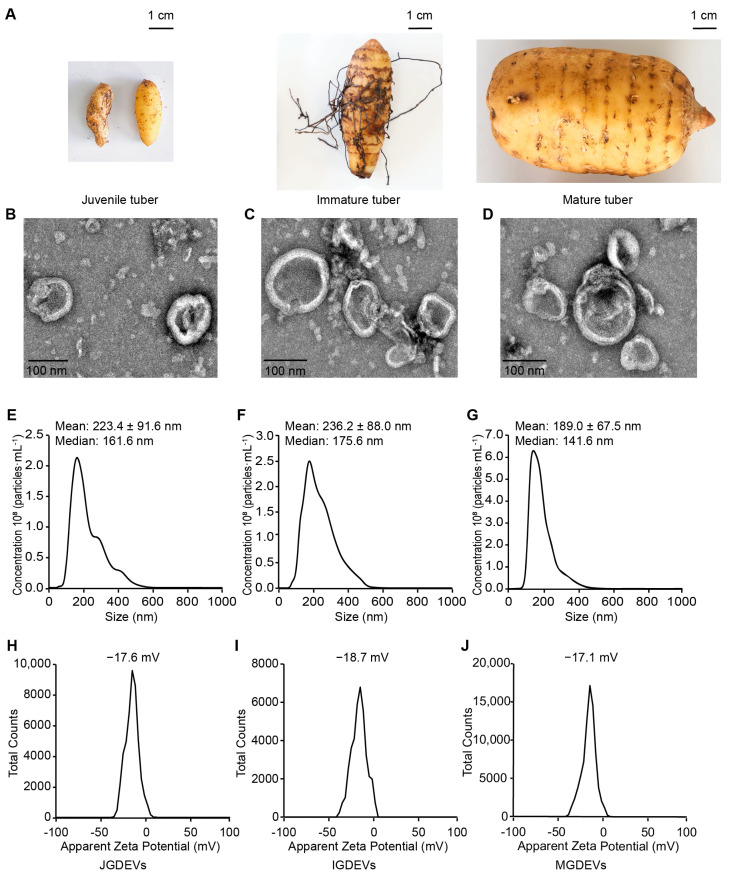
Characterization of *G. elata*-derived extracellular vesicles (GDEVs) across different developmental stages. (**A**) Morphological characterization of *G. elata* tubers at distinct developmental phases, juvenile tuber, immature tuber, and mature tuber, from left to right. (**B**–**D**) TEM images of JGDEVs, IGDEVs, and MGDEVs. (**E**–**G**) Particle size distribution of JGDEVs, IGDEVs, and MGDEVs. (**H**–**J**) Zeta potential of JGDEVs, IGDEVs, and MGDEVs.

To investigate the characteristics of GDEVs from different developmental stages, vesicles were isolated from three representative developmental stages: juvenile tuber, immature tuber, and mature tuber. The physical morphology of tubers at each stage is shown in Figure 1A. Juvenile tubers were observed as small, globular or oval structures, while the surface of immature tubers was characterized by abundant *Armillaria* mycelia, the symbiotic fungus essential for the growth of *G. elata*. As the tubers matured, they increased in size and developed a distinct morphology; notably, mature tubers no longer sustained symbiosis with *Armillaria*.

GDEVs were successfully isolated from each developmental stage using differential centrifugation and ultracentrifugation methods. The ultrastructure of these vesicles was subsequently examined by transmission electron microscopy (TEM). As depicted in Figure 1B–D, GDEVs derived from juvenile (B), immature (C), and mature (D) tubers exhibited typical cup-shaped or spheroidal bilayer membrane structures, which are consistent with the general morphological features of plant extracellular vesicles.

NTA was further conducted to determine the particle size distribution and the concentration of extracellular vesicles across the three developmental stages (Figure 1E–G). Juvenile (JGDEVs), immature (IGDEVs), and mature tuber-derived vesicles (MGDEVs) displayed mean diameters of 223.4 ± 91.6 nm, 236.2 ± 88.0 nm, and 189.0 ± 67.5 nm, respectively. The majority of GDEVs ranged from approximately 100 to 400 nm in diameter.

To assess vesicle surface charge, the zeta potential of JGDEVs, IGDEVs, and MGDEVs was analyzed (Figure 1H–J). The apparent zeta potential values were −17.6 mV for JGDEVs, −18.7 mV for IGDEVs, and −17.1 mV for MGDEVs, suggesting that all vesicle populations carried a comparable moderately negative surface charge, indicative of good colloidal stability.

Collectively, these results demonstrate that vesicles can be successfully isolated from *G. elata* tubers at different developmental stages, and consistently exhibit a typical extracellular vesicle morphology, a sub-micron particle size distribution, and stable surface charge properties.

### 2.2. Comprehensive Lipidomic Profiling Reveals Metabolic Shifts Across G. elata Tuber Development

The lipidomic profiling of GDEVs at three developmental stages, namely JGDEV, IGDEV, and MGDEV, revealed substantial variations in lipid composition. Initially, a total of 3412 lipid molecules were identified across all samples (Table S1) and categorized into eight major classes based on the LIPID MAPS system: fatty acyls (FAs), glycerolipids (GLs), glycerophospholipids (GPs), sphingolipids (SPs), sterol lipids (STs), prenol lipids (PRs), saccharolipids (SLs), and polyketides (PKs) [18]. To ensure robustness and biological relevance, only compounds with an abundance greater than 100 ppm in at least one sample were retained, resulting in a final dataset of 996 lipid species for subsequent analyses (Table S2). This filtering step excluded low-abundance or potentially artifactual signals, thus enhancing the reliability of downstream quantitative and multivariate statistical analyses.

**Figure 2:**
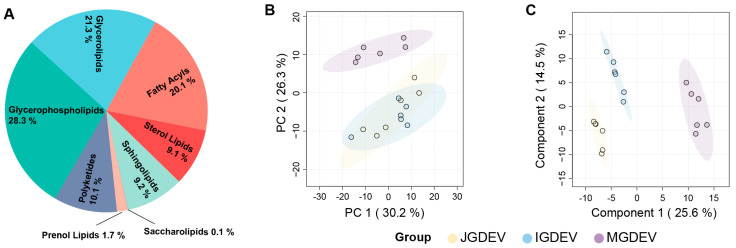
Lipidomic profiling reveals altered lipid metabolism among different *G. elata* developmental stages. (**A**) The composition of lipid categories; (**B**) Principal component analysis (PCA) score plot; (**C**) partial least squares discriminant analysis (PLS-DA) score plot.

Based on the filtered dataset, the GDEV lipidome comprises 28.3% GPs, 21.3% GLs, 20.1% FAs, 10.1% PKs, 9.1% STs, and 9.2% SPs, in addition to minor fractions of PRs (1.7%) and SLs (0.1%) (Figure 2A, Table S3). To further elucidate stage-specific lipidomic alterations, multivariate statistical analyses were conducted. Principal component analysis (PCA) revealed a clear separation between MGDEVs and those from earlier stages (JGDEVs and IGDEVs) along the first two principal components, which together explained 30.2% and 26.3% of the total variance, respectively. However, the PCA showed a high degree of overlap between JGDEV and IGDEV, indicating limited lipidomic divergence between these two early developmental stages (Figure 2B). In contrast, partial least squares discriminant analysis (PLS-DA) achieved clear discrimination among all three groups, with components 1 and 2 explaining 25.6% and 14.9% of the variance, respectively, underscoring significant lipidomic remodeling during tuber maturation (Figure 2C).

### 2.3. ANOVA-Based Identification and Clustering of Stage-Associated Lipids

**Figure 3:**
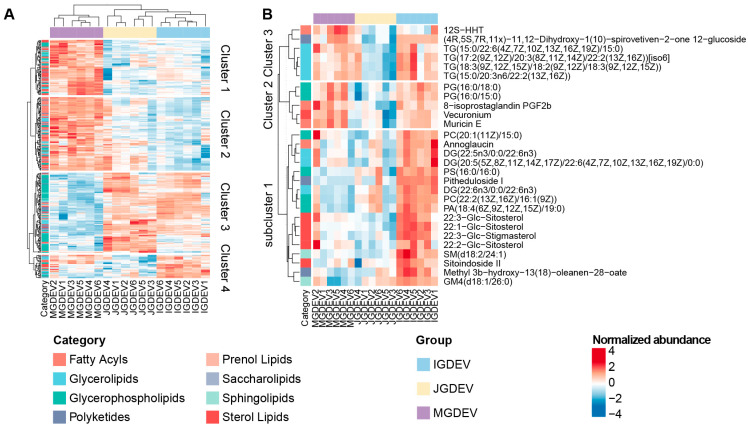
Lipidomic profiling of extracellular vesicles from *Gastrodia elata* tubers at different developmental stages. (**A**) Hierarchical clustering heatmap showing significantly altered lipid species in extracellular vesicles isolated from juvenile (JGDEV), immature (IGDEV), and mature (MGDEV) tubers (n = 6 biological replicates per stage). Rows correspond to individual lipid species; columns represent sample replicates. Relative abundance is expressed as z-score and color-graded from blue (low) to red (high). Five major lipid clusters are demarcated and annotated on the right. Key lipid markers are emphasized with bold labels. (**B**) Subcluster analysis of lipid cluster 3, highlighting three abundance patterns across developmental stages. Representative lipids and their respective chemical classes are indicated.

To further elucidate the lipidomic dynamics during tuber development, ANOVA was performed on the filtered set of 996 lipid species detected in GDEVs. A total of 278 lipids were identified, exhibiting statistically significant differences among the three developmental stages. The hierarchical clustering of these stage-associated lipids resolved five distinct clusters, each displaying coordinated regulation and characteristic patterns across developmental stages (Figure 3A).

Clusters 1 and 2 were mainly upregulated in MGDEVs, with cluster 2 displaying a relatively higher abundance in JGDEVs compared with cluster 1. Notably, fatty acids were significantly enriched in cluster 2 (Fisher’s exact test, *p* < 0.05), suggesting enhanced fatty acid metabolism associated with these developmental stages. Cluster 3 was characterized primarily by GPs and showed an elevated abundance in both IGDEVs and JGDEVs, highlighting the importance of this lipid class during tuber development and early symbiotic events.

Cluster 4 included lipid species that were specifically accumulated in IGDEVs, aligning with the stage of maximal symbiotic interaction with *Armillaria* and implicating potential roles in lipid-mediated plant–microbe signaling processes. Given the central role of the immature tuber stage in the symbiotic association with *Armillaria*, we further investigated cluster 4 in detail. The further clustering of cluster 4 revealed three subclusters with distinct expression profiles (Figure 3B). Among these, subcluster 1 exhibited a pronounced and specific upregulation in IGDEVs. This subcluster comprised 17 lipid species, notably including 4 Glc-Sitosterol derivatives and 3 diacylglycerols (DGs).

### 2.4. OPLS-DA Reveals Major Lipidomic Differences Across Developmental Stages

To further elucidate stage-specific lipidomic remodeling during tuber development, we conducted an orthogonal partial least squares discriminant analysis (OPLS-DA) to compare the lipid profiles of IGDEVs with those of both MGDEV and JGDEV samples.

**Figure 4:**
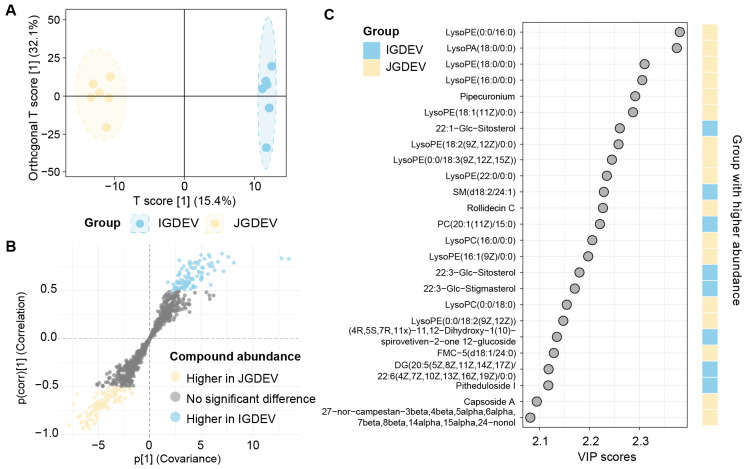
OPLS-DA analysis of extracellular vesicle lipids from *G. elata* tubers at juvenile (JGDEV) and immature (IGDEV) stages. (**A**) OPLS-DA score plot; (**B**) S-plot; (**C**) variable importance in projection (VIP) scores.

The OPLS-DA model achieved a clear separation between IGDEV and JGDEV groups (Figure 4A), with the first predictive component explaining 15.4% of total X variance and 87.8% of Y variance (Q^2^ = 0.489, Table S4). The model incorporated three orthogonal components (o1–o4), which together accounted for 89.3% of X variance unrelated to group separation (cumulative R^2^Y = 0.118), suggesting substantial within-group variance distinct from the group-discriminating axis. Overall, the model parameters showed excellent explanatory power (cumulative R^2^X = 89.3%, R^2^Y = 99.5%, Q^2^ (cum) = 0.919; Table 1), further validated by permutation tests (n = 100; Figure S1). To identify candidate biomarkers driving group separation, S-plot analysis was performed. A total of 209 variables were identified as potential discriminatory metabolites, with 94 variables showing a significantly higher abundance in IGDEV, while 115 were more abundant in JGDEV (Figure 4B). Further variable importance in projection (VIP) analysis highlighted 342 compounds (VIP > 1) (Table S5), among which 136 showed a higher abundance in IGDEV. The top 25 compounds by VIP score included distinct lysophosphatidylethanolamines (LysoPEs) and Glc-sitosterols (Figure 4C).

**Figure 5:**
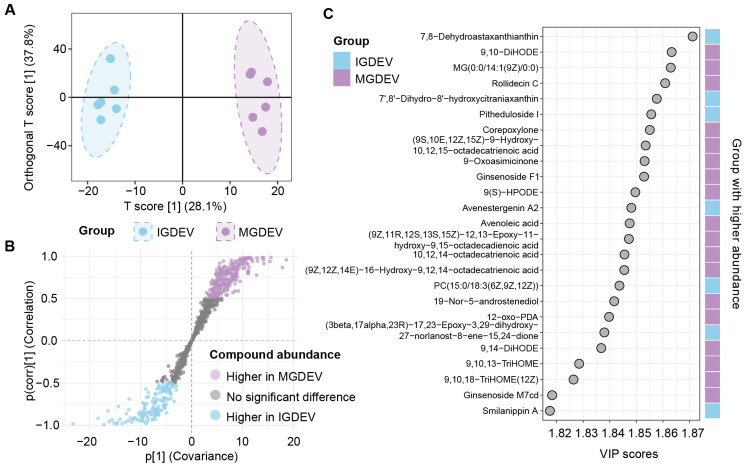
OPLS-DA analysis of extracellular vesicle lipids from *G. elata* tubers at juvenile (MGDEV) and immature (IGDEV) stages. (**A**) OPLS-DA score plot; (**B**) S-plot; (**C**) variable importance in projection (VIP) scores.

Similarly, the OPLS-DA also achieved clear group separation for the IGDEV vs. MGDEV comparison (Figure 5A, Figure S2). The first predictive component (p1) explained a substantial proportion of the group variance, with 28.1% of X variance (R^2^X = 0.281) and 94.4% of Y variance (R^2^Y = 0.944), and a predictive ability (Q^2^) of 0.713. Unlike the IGDEV vs. JGDEV comparison, which included three orthogonal components, this model required only two. The two orthogonal components explained an additional 37.8% (o1: 0.378) and 13.1% (o2: 0.131) of X variance, respectively (Table S6). Cumulatively, the model explained 79.1% of X variance and 99.1% of Y variance, with Q^2^(cum) = 0.927 (Table 1). The S-plot analysis (Figure 5B) identified 401 differentially abundant variables, with 262 compounds elevated in MGDEV and 139 in IGDEV. The VIP analysis identified 379 compounds with scores greater than 1, of which 131 were elevated in IGDEV (Table S7). Notably, metabolites such as 7,8-dehydroastaxanthin, 7′,8′-Dihydro-8′-hydroxycitraniaxanthin, pitheduloside I, and avenestergenin A2 were among those with a higher abundance in IGDEV (Figure 5C).

### 2.5. Quantitative Differential Analysis of Key Lipid Species

**Figure 6:**
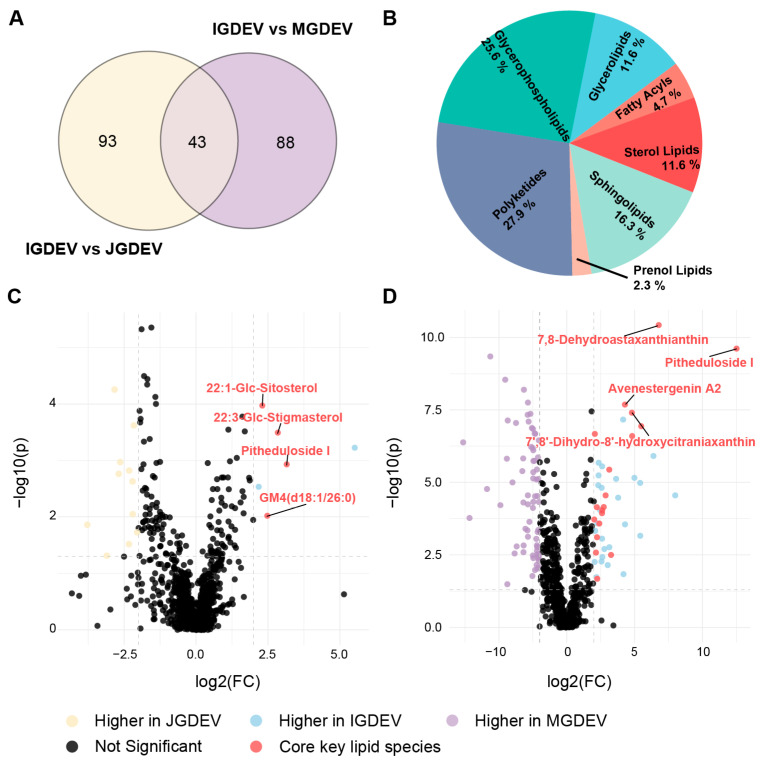
Quantitative differential analysis of key lipid species among GDEVs at different developmental stages. (**A**) Venn diagram showing the overlap of significantly changed lipid species identified by OPLS-DA between IGDEV vs. JGDEV and IGDEV vs. MGDEV comparisons, with 166 species shared between the two groups. (**B**) Lipid class distribution of these 166 shared differentially accumulated lipid species. Volcano plots showing quantitative differences in lipid species abundance between IGDEV and JGDEV (**C**), and IGDEV and MGDEV (**D**). Yellow, blue, and purple indicate lipid species with a significantly higher abundance in JGDEV, IGDEV, and MGDEV, respectively; black dots indicate non-significant differences, and red dots highlight core key lipid species.

A total of 43 metabolites were consistently identified as differentially accumulated in IGDEVs in both the IGDEV vs. JGDEV and IGDEV vs. MGDEV comparisons using OPLS-DA and are hereafter referred to as core lipid markers of the symbiotic stage (Figure 6A; Table S8). These 43 core lipid markers were significantly enriched in polyketides (Fisher’s exact test, *p* < 0.05; Figure 6B).

To comprehensively assess the magnitude of lipidomic alterations during tuber maturation, we evaluated the fold changes for all detected lipid species (Figure 6C,D). In the IGDEV vs. JGDEV comparison (Figure 6C), only 17 lipid species showed significant alterations. Among these, four core lipid markers were markedly upregulated in IGDEV, namely 22:1-Glc-Sitosterol, 22:3-Glc-stigmasterol, pitheduloside I, and GM4 (d18:1/26:0) with log_2_FC values of 2.30, 2.84, 3.15, and 2.48, respectively, underscoring their pivotal accumulation during the initiation of mycorrhizal symbiosis (Table S9). Conversely, eleven compounds were downregulated in IGDEV relative to JGDEV, with log_2_FC values between −2.05 and −3.78 (Table S8).

Similar patterns were observed in the IGDEV vs. MGDEV comparison (Figure 6D), where 114 lipid species displayed significant abundance shifts (Table S10). Specifically, 7,8-dehydroastaxanthin and pitheduloside I showed strong upregulation in IGDEV (log_2_FC = 6.78 and 12.52, respectively), suggesting their role as symbiosis-stage specific metabolic markers.

## 3. Discussion

### 3.1. Plant-Derived Extracellular Vesicles as Novel Mediators in Mycoheterotrophic Symbiosis

*G. elata* is a fully mycoheterotrophic orchid that relies exclusively on a symbiotic association with *Armillaria* during its subterranean vegetative growth. Throughout the vegetative growth phase, the tubers of *G. elata* undergo three distinct developmental stages: juvenile, immature, and mature [1]. At the juvenile stage, tubers establish initial contact with *Armillaria* mycelia, forming early symbiotic interfaces essential for nutrient acquisition. The immature stage is characterized by ongoing fungal colonization and maximal nutrient transfer at the plant–fungus interface. In contrast, at the mature stage, tubers shift to accumulating reserves and preparing for subsequent reproductive development, during which the symbiotic relationship with *Armillaria* is gradually diminished and extensive mycelial colonization is no longer observed.

In this study, PDEVs were identified from *G. elata* tubers at juvenile, immature, and mature developmental stages, expanding our understanding of the distribution and conservation of PDEVs across both species and tissue types. Notably, EVs were successfully isolated from orchid tubers, which are structurally distinct from roots and leaves that have been the primary focus of previous plant EV research. This finding demonstrates that EVs are not only widespread across various plant species, but are also present in diverse organs, including the previously unexplored tubers of Orchidaceae.

GDEVs exhibited classical cup-shaped or spheroidal structures and sub-micron particle sizes, in agreement with the canonical characteristics reported for plant EVs in *Arabidopsis thaliana* [19], sunflower (*Helianthus annuus*) [14], and grapefruit (*Citrus* × *paradisi*) [20]. The moderately negative zeta potential observed for these vesicles indicates colloidal stability, which may facilitate their movement within the dense tuber matrix and support effective interactions with symbiotic fungi.

Importantly, the consistent presence of EVs across all developmental stages examined suggests that their occurrence is a general feature not restricted to specific tissues or growth phases. These observations provide new evidence for the broad distribution and potential physiological significance of plant-derived EVs and lay the groundwork for further investigation into their roles in symbiotic communication and metabolic coordination within mycoheterotrophic systems.

### 3.2. Lipidomic Remodeling Reflects Developmental Transitions and Symbiotic Interactions

The comprehensive lipidomic analysis of GDEVs revealed remarkable chemical diversity, with 996 lipid species detected across all major lipid categories. The number of lipid species identified in GDEVs falls within the range reported for PDEVs from other plant species. For example, *Arabidopsis* leaf EVs contain approximately 300 lipid species [19], ginger (*Zingiber officinale*)-derived EVs (GEVs) contain around 1300 [21], and lemon (*Citrus* × *limon*)-derived EVs (LEVs) have about 1200 [22].

Apart from a comparable number of lipid species, GDEVs exhibit high compositional diversity and are distinguished by a more even distribution of lipid classes compared with previously reported PDEVs. For example, whereas grapefruit-derived EVs are dominated by GPs (50.05% of total lipids), and ginger-, lemon-, and grape-derived EVs are characterized by a predominance of GLs (46.23–53.77%) [22], GDEVs present a notably balanced lipid profile. In GDEVs, GPs remain the largest category but only comprise 28.3% of the total lipids, closely followed by GLs at 21.3%. This absence of a single dominant lipid class distinguishes GDEVs from most other PDEVs reported to date. Such balanced lipid partitioning may reflect the unique metabolic strategies of *G. elata* in sustaining a fully mycoheterotrophic lifestyle, as well as characteristics shared with other Orchidaceae. Previous studies have indicated that the high similarity in lipid composition between GFEVs and LEVs might be associated with their common origin from the *Citrus* genus [22].

To further evaluate the lipidomic differences among the developmental stages of GDEVs, both unsupervised principal component analysis (PCA, Figure 2B) and supervised partial least squares discriminant analysis (PLS-DA, Figure 2C) were performed on the 996 filtered lipid species. The PCA revealed that while MGDEV samples formed a distinct cluster, IGDEV and JGDEV samples showed considerable overlap, indicating limited variance between these early stages. In contrast, the PLS-DA robustly resolved all three groups, clearly distinguishing the lipidomes of juvenile, immature, and mature tuber-derived EVs. This enhanced sensitivity of supervised approaches underscores the presence of discrete, stage-specific lipidomic signatures, particularly between the juvenile and immature stages.

These findings indicate that the vesicular lipidome of *G. elata* undergoes developmentally regulated remodeling, likely to support the changing functional requirements of tuber maturation and symbiotic interaction. Similar stage-specific lipid changes have been observed in *Lotus japonicus*, where the abundance of palmitvaccenic (di-16:1) or tetracosenoic (24:1) acyl groups, two FAs, decrease in intraradical mycelium after mycorrhization information [23]. This dynamic lipid remodeling highlights the importance of identifying the key molecular components in EVs derived from immature tubers, which represent the critical stage for symbiosis establishment.

### 3.3. Core Lipid Markers in GDEVs and Their Potential Roles in Symbiotic Signaling

To pinpoint the lipid species that may mediate symbiotic signaling during the critical immature tuber stage, we integrated unsupervised clustering analysis on compounds filtered by ANOVA, alongside supervised modeling (OPLS-DA).

Unsupervised clustering revealed a cluster of 17 compounds highly expressed in IGDEVs, in which four Glc-sitosterols were particularly notable (Figure 3B). Glc-sitosterol belongs to the steryl glycosides class [24]. Plant sterols typically exist in three forms, free sterols, sterol esters, and steryl glycosides, with Glc-sitosterol representing the glycosylated form [25]. Previous research has primarily focused on its biosynthesis [25] and its structural function within plant membranes [24].

Recent studies have highlighted the active role of plant-derived sterols in modulating microbial behavior during plant–microbe interactions [26,27]. In the *Arabidopsis*–*Pseudomonas* model, *A. thaliana* utilizes the enzyme CYP710A1 to convert β-sitosterol to stigmasterol. The accumulation of stigmasterol has been demonstrated to inhibit the growth of the pathogenic bacterium *Pseudomonas syringae*, thereby enhancing plant defense [27]. This exemplifies how plants dynamically adjust their sterol composition to actively constrain pathogen proliferation.

To further prioritize candidate lipids, we complemented our clustering analysis with OPLS-DA, a supervised method with superior discriminatory capability in our dataset. A total of 43 core lipid markers were consistently found to be significantly enriched in IGDEVs compared with both MGDEVs and JGDEVs (Table S8). These 43 compounds are predominantly classified as PKs (Figure 6A). PKs are a diverse class of secondary metabolites biosynthesized via the condensation of acetyl-CoA and malonyl-CoA units by polyketide synthases [28]. They exhibit a broad range of biological activities, including antimicrobial, antifungal, and signaling properties [29]. In plants, PKs are involved in various ecological interactions, functioning as defensive compounds or signaling molecules to mediate plant–microbe relationships. Recent studies indicate that PKs play essential roles in facilitating symbiotic interactions. For instance, plants produce C-glycosylated aromatic polyketides that serve as substrates for beneficial symbiotic bacteria like *Deinococcus aerius*. These bacteria deglycosylate the compounds, releasing the active aglycone form, which then promotes beneficial functions such as facilitating nitrogen fixation or acting as antibacterial agents against pathogens, thus enhancing plant health and symbiosis [30].

In this study, 7,8-dehydroastaxanthin was identified as the most important PK-type core lipid marker in the IGDEV. 7,8-Dehydroastaxanthin, a carotenoid with a polyketide backbone [31], has not yet been directly implicated in plant–microbe interactions. Nevertheless, carotenoids derived from bacteria, yeasts, algae, and plants are recognized for their powerful reactive oxygen species (ROS) scavenging capabilities, including the mitigation of ROS generated by fungal pathogens [32]. In *G. elata*–*Armillaria gallica* symbiosis, the maintenance of ROS homeostasis has been shown to play a pivotal role in symbiotic signaling [33]. Thus, it is reasonable to speculate that 7,8-dehydroastaxanthin may influence the interaction between *G. elata* and *Armillaria* by modulating ROS levels.

Both sets of core lipid markers identified by the unsupervised and supervised approaches were confirmed by subsequent quantitative analyses (Figure 6, Table S8). These findings collectively indicate that Glc-sitosterol and 7,8-dehydroastaxanthin are among the most important lipid mediators transported via PDEVs during *G. elata*–*Armillaria* symbiosis. Although other regulatory pathways (e.g., phytohormones or rhizosphere secretions) may also play significant roles, our results suggest that these two lipids are specifically enriched in PDEVs and may represent core mediators of symbiotic communication through vesicle-based signaling.

### 3.4. Future Perspectives

While this study establishes the developmental and metabolic plasticity of GDEV lipidomes, significant questions remain regarding the full spectrum of their molecular cargo and their roles in *G. elata*–*Armillaria* symbiosis. While this study reveals the developmental and metabolic dynamics of GDEV lipidomes, further studies are necessary to examine whether GDEVs’ lipidome is influenced by fungal factors such as mycelial activity or *Armillaria* species, and to characterize the full spectrum of GDEVs’ molecular cargo. Notably, in *A. thaliana*, exposure to two fungal pathogens within the same genus, *Colletotrichum higginsianum* and *Colletotrichum destructivum*, triggers the production of EVs with distinct molecular compositions [11]. This suggests that similar species-specific responses may operate in mycoheterotrophic systems such as *G. elata*, which depends completely on fungal partners for carbon and energy. Previous studies have indicated that *G. elata* can form symbiotic relationships with different *Armillaria* species of differing pathogenicity [34]; therefore, comparative analyses of GDEVs across different *Armillaria* species and states of mycelial activity are needed to test the influence of fungal identity and physiology on lipid composition.

Beyond lipids, mounting evidence from AM and other plant symbiotic systems indicates that PDEVs are also enriched in diverse functional proteins, such as small secreted effectors, cell wall-modifying enzymes, and membrane-trafficking components [9,35]. Notably, certain small secreted proteins have been identified exclusively in AM host plants and may be specifically trafficked to symbiotic interfaces via EVs [36]. Vesicle-associated membrane proteins, for example, VAMP721s, have been linked to enzyme delivery at symbiotic boundaries in root nodules [35], and similar mechanisms may exist during the *Armillaria* colonization of *G. elata*. Therefore, the comprehensive proteomic profiling of GDEVs across developmental stages will be crucial to reveal symbiosis-specific protein cargos, clarify their trafficking pathways, and determine their functional significance in fungal accommodation, immune modulation, or cell wall remodeling.

In addition to proteins, the potential for GDEVs to mediate small RNA and miRNA transfer is a particularly compelling avenue for future research. In both mutualistic and pathogenic plant–microbe interactions, EVs act as vehicles for small RNAs, enabling cross-kingdom gene regulation and RNA interference [14,37,38]. In arbuscular mycorrhizal symbioses, small RNAs from both host plants and fungi can function as mobile signals, suggesting sophisticated RNA-level dialog [39]. However, whether and how GDEVs encapsulate and transport regulatory RNAs to *Armillaria*, and the outcomes of such molecular exchanges, is unknown in the mycoheterotrophic context. The detailed characterization of the miRNA and sRNA profiles of GDEVs, as well as the identification of their potential fungal targets, will provide key insights into the mechanisms of RNA-based communication, defense, and the establishment of mutualistic compatibility.

In summary, integrative proteomic and small RNA analyses, as well as investigations into EV heterogeneity, will be essential for a holistic understanding of GDEV function and for unraveling the molecular mechanisms underlying this unique plant–fungal symbiosis.

## 4. Materials and Methods

### 4.1. Isolation and Characterization Purification of GDEVs from Different Developmental Phases

GDEVs were isolated from *G. elata* tubers (Yunnan Province, China). The tubers were grown in symbiosis with *Armillaria gallica*, following previously described detailed cultivation methods [40]. Prior to EV isolation, all associated *A. gallica* mycelium was meticulously removed from the tubers using the same method employed in our previous studies for preparing *G. elata* samples for detection assays [41]. GDEVs were then isolated using a differential centrifugation protocol with modifications [42]. The isolation procedure was performed for tubers at three developmental stages: mature (MGDEVs), immature (IGDEVs), and juvenile (JGDEVs). Fresh tubers were washed thoroughly with deionized water and homogenized in PBS buffer (1:1 *v*/*v*) using a blender. The homogenate was sequentially centrifuged at 4000×*g* for 40 min and 10,000× *g* for 60 min at 4 °C to remove cellular debris. The cleared supernatant was then subjected to ultracentrifugation at 120,000× *g* for 2 h (4 °C) to collect the EV-enriched pellet. For further purification, the pellet was resuspended in PBS and layered onto a discontinuous sucrose gradient (8%, 30%, 45%, and 60% *w*/*v*) followed by ultracentrifugation at 120,000× *g* for 16 h at 4 °C. The fraction containing purified GDEVs was collected from the 30–45% sucrose interface. Protein concentrations were determined using a BCA protein assay, and all GDEVs preparations were stored at −80 °C until further analysis.

### 4.2. Transmission Electron Microscopy (TEM) Imaging

TEM analysis was conducted on an FEI Tecnai G2 Spirit microscope (FEI company, Hillsboro, OR, USA) using negative staining. Briefly, purified GDEVs in 5 μL aliquots were loaded onto formvar-coated 200-mesh copper grids and incubated for 1 min at room temperature to facilitate adsorption.

### 4.3. Nanoparticle Tracking Analysis (NTA)

The concentration and size distribution of GDEVs were analyzed using nanoparticle tracking analysis (NanoSight NS300, Malvern Instruments Ltd., Malvern, UK). Purified GDEVs were diluted 1:100 in PBS to achieve an optimal particle concentration of 10^8^–10^9^ particles/mL. Measurements were conducted at 25 °C using a 488 nm blue laser module. Particle trajectories were recorded in triplicate and analyzed using NTA 3.4 software (Malvern Panalytical Ltd., Malvern, UK) with a detection threshold optimized for low-refractive-index biological nanoparticles.

### 4.4. Zeta Potential Analysis

The surface charge of GDEVs was determined by zeta potential measurements using dynamic light scattering (Zetasizer Nano ZS, Malvern Panalytical Ltd., Malvern, UK). Purified GDEVs were diluted in 10 mM PBS (pH 7.4) to a final concentration of 10^8^ particles/mL and loaded into a disposable folded capillary cell. Measurements were performed at 25 °C. Data were processed using Zetasizer Software 7.13.

### 4.5. Lipidomics Analysis

For the lipidomic analysis of plant vesicles, equal amounts of MGDEV, IGDEV, and JGDEV samples were processed using a modified methyl tert-butyl ether (MTBE) extraction method. Briefly, each vesicle sample was mixed with 300 μL methanol and 1000 μL MTBE, followed by vigorous vortexing and sonication for 1 h. After adding 250 μL deionized water, the mixture was vortexed thoroughly and centrifuged at 12,000× *g* for 5 min at 4 °C. The upper organic phase (400 μL) was carefully collected in a 1.5 mL tube, dried under vacuum, and subsequently reconstituted in 100 μL acetonitrile–isopropanol (1:1, *v*/*v*) solution for LC-MS/MS analysis.

Quality control (QC) samples were prepared by pooling equal aliquots of all experimental samples, ensuring a representative lipid composition. These QC samples underwent identical processing and analytical procedures as the test samples. This approach allowed for monitoring system stability and reproducibility throughout the LC-MS/MS analyses.

Lipid profiling was performed using a quadrupole time-of-flight mass spectrometer (Q Exactive, Thermo Scientific, Waltham, MA, USA) coupled with an UltiMate™ 3000 RSLCnano system (Thermo Scientific, Waltham, MA, USA). Chromatographic separation was achieved on a Waters Acquity UPLC BEH C8 column (1.7 µm, 2.1 mm ×100 mm) with a flow rate of 0.25 mL/min. The mobile phase consisted of (A) acetonitrile/water (60:40, *v*/*v*) and (B) isopropanol/acetonitrile (90:10, *v*/*v*), both containing 10 mmol/L ammonium formate and 0.1% formic acid. The gradient program was as follows: 98% B (0–1 min), 98–30% B (1–5 min), 30–0% B (5–8 min), 0% B (8–14 min), and re-equilibration to 98% B (14–16 min). Mass spectrometry detection operated in dual-polarity mode (positive/negative HESI) with the following parameters: spray voltage ± 2.5 kV, sheath/auxiliary gas 30/10 psi, capillary temperature 320 °C, scan range 100–1500 *m*/*z*, and MS1/MS2 resolutions 70,000/17,500 FWHM (*m*/*z* 200).

Raw LC-MS/MS data were processed using Progenesis QI 2.3 (Waters) for feature detection, alignment, and annotation. Peak filtering criteria included mass error < 5 ppm, CV < 30% across replicates. Lipid identification was performed by matching accurate mass (±5 ppm) and MS/MS fragmentation patterns against the LIPID MAPS^®^ database, HMDB, and PubChem database. Only lipids detected in ≥75% of biological replicates (≥3/4 samples per group) with quantifiable signals were retained. Lipid classification in categories, main class, and subclass was carried out according to the LIPID MAPS^®^ database [43].

### 4.6. Statistical Analysis

Statistical analyses were performed using MetaboAnalyst 6.0 [44]. Raw peak intensities were normalized to the total peak area to minimize technical variation. The normalized data were log10-transformed and Pareto-scaled prior to downstream analysis. Principal component analysis (PCA) was first conducted to visualize the overall metabolic differences among samples. Partial least squares discriminant analysis (PLS-DA) was subsequently applied to maximize group separation and identify the metabolites contributing most to intergroup variation.

For univariate analysis, a one-way analysis of variance (ANOVA) was conducted to identify the significantly different metabolites among experimental groups. Hierarchical clustering analysis and heatmap visualization were performed based on the ANOVA results, employing Euclidean distance and Ward’s linkage (ward.D) methods, to illustrate the relative abundance patterns of significant metabolites across groups. Multiple testing correction was conducted using the Benjamini–Hochberg method to control the false discovery rate (FDR, *p* < 0.05). For pairwise comparisons (e.g., IGDEV vs. JGDEV), significantly altered lipids were identified using an FDR-adjusted t-test (Benjamini–Hochberg, *p* < 0.05) with a fold-change cutoff (|log_2_FC| > 2) for biological relevance [45].

To further investigate group-specific metabolic distinctions, OPLS-DA was carried out. OPLS-DA enabled the separation of predictive (between-group) and orthogonal (within-group) variation, thereby improving model interpretability. Model performance was evaluated using R^2^Y (explained variance) and Q^2^ (predictive ability) values, with model validation conducted via permutation testing (n = 100) to assess the risk of overfitting. The criteria for selecting marker metabolites were defined in accordance with previous reports [46]. VIP scores derived from the OPLS-DA model were used to rank metabolites according to their influence on group discrimination, with VIP > 1 considered as the threshold for key contributors. Additionally, candidate discriminatory metabolites were identified from the S-plot derived from the OPLS-DA model using threshold values of absolute covariance [|p(1)| ≥ 0.05] and correlation [|p(corr)(1)| ≥ 0.5].

All statistical analyses followed recommended procedures for untargeted metabolomics and were performed in triplicate (n = 6 per group). Data visualization and result exportation were carried out using MetaboAnalyst 6.0 and R version 4.5.0, unless otherwise specified.

## 5. Conclusions

This study establishes a comprehensive lipidomic profile of GDEVs across three key tuber developmental stages, confirming our initial hypothesis that GDEV lipid composition not only varies significantly during tuber maturation but also distinctly differs from that of photosynthetic species. Furthermore, the lipid profile of GDEVs is dynamically remodeled in a stage-specific manner, closely associated with symbiotic activity. Notably, immature tubers exhibited a significant enrichment of lipids such as Glc-sitosterols and 7,8-dehydroastaxanthin. This lipid accumulation pattern provides strong support for our second hypothesis, indicating that specific lipid classes play essential roles in symbiotic interactions. Based on these findings, we propose that these lipids may serve as key signaling molecules delivered via GDEVs to modulate fungal interaction, though their precise functions require further functional validation. Overall, this study lays a solid foundation for unraveling the molecular mechanisms of plant–fungal communication in mycoheterotrophic systems and offers a new framework for exploring lipid-mediated signaling in non-model plant–microbe interactions. Future studies integrating multi-omics approaches, such as the proteomic and small RNA profiling of GDEVs, will be essential to fully elucidate the repertoire of EV cargoes orchestrating the symbiosis between *G. elata* and *Armillaria*.

## Figures and Tables

**Table 1:** Statistics of OPLS-DA model performance for group comparisons.

Model	R^2^X (cum)	R^2^Y (cum)	Q^2^ (cum)
IGDEV–JGDEV	0.893	0.995	0.919
IGDEV–MGDEV	0.991	0.991	0.927

R^2^X (cum) represents the cumulative explained variance of X-variables by all components in the model; R^2^Y (cum) represents the cumulative explained variance of Y-variables by all predictive components.; Q^2^ (cum) indicates the cumulative predictive ability of the model evaluated by cross-validation.

## Data Availability

The original contributions presented in this study are included in the article/Supplementary Materials. Further inquiries can be directed to the corresponding authors.

## Supplementary Materials

The following supporting information can be downloaded at: https://www.mdpi.com/article/10.3390/ijms26178611/s1.

## Abbreviations

The following abbreviations are used in this manuscript:

AM	arbuscular mycorrhizal
EVs	extracellular vesicles
FAs	fatty acyls
GDEVs	*G. elata*-derived extracellular vesicles
GL	glycerolipid
GP	glycerophospholipid
IGDEV	immature tuber-derived extracellular vesicle
JGDEV	juvenile tuber-derived extracellular vesicle
MGDEV	mature tuber-derived extracellular vesicle
NTA	nanoparticle tracking analysis
OPLS-DA	orthogonal partial least squares discriminant analysis
PCA	principal component analysis
PDEVs	plant-derived extracellular vesicles
PK	polyketide
PLS-DA	partial least squares discriminant analysis
PR	prenol lipid
SL	saccharolipid
SP	sphingolipid
ST	sterol lipid
TEM	transmission electron microscopy
