# Supplementary material for: Stage-Specific Lipidomes of Gastrodia elata Extracellular Vesicles Modulate Fungal Symbiosis
Source: Int J Mol Sci. 2025 Sep 4;26(17):8611. doi: 10.3390/ijms26178611 (PMC12429340; doi:10.3390/ijms26178611)
Supplement: Supplementary file 1 [file ijms-26-08611-s001.zip › Supplementary Figures.pdf]

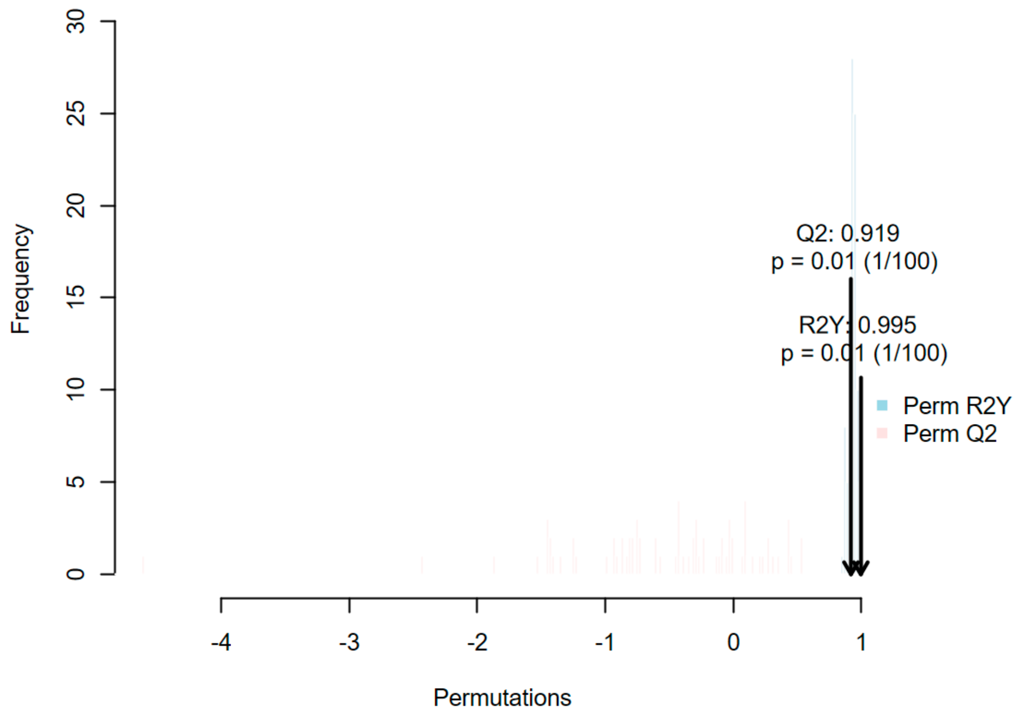

Figure S1 Permutation test validating the OPLS-DA model for JGDEV vs. IGDEV groups.

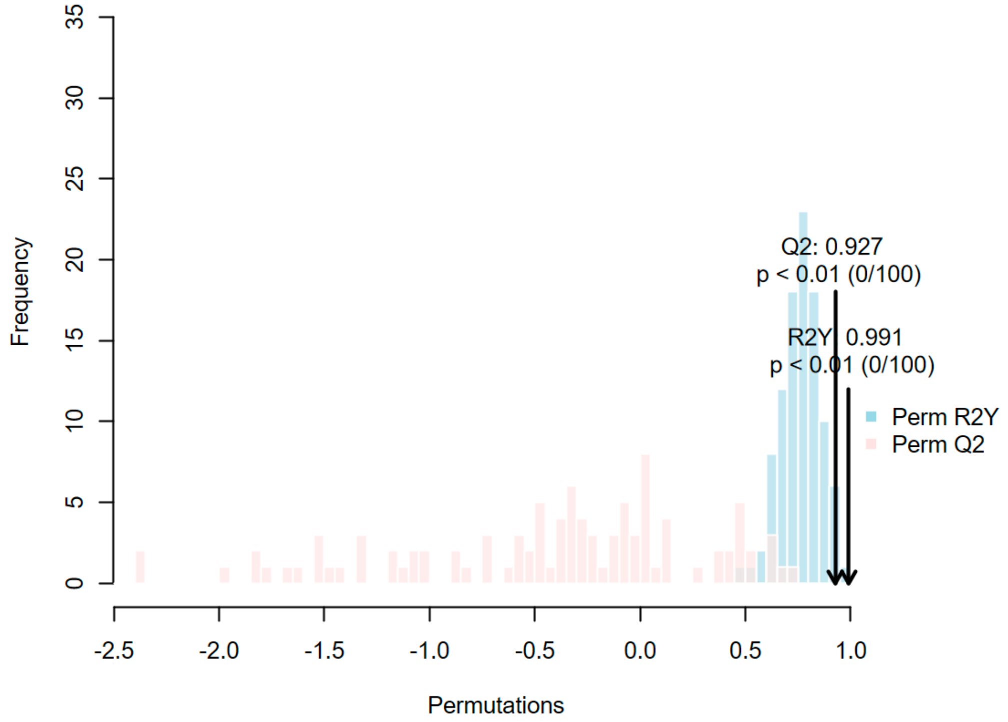

Figure S2 Permutation test validating the OPLS-DA model for IGDEV vs. MGDEV groups.
